# M1 Macrophage and M1/M2 ratio defined by transcriptomic signatures resemble only part of their conventional clinical characteristics in breast cancer

**DOI:** 10.1038/s41598-020-73624-w

**Published:** 2020-10-06

**Authors:** Masanori Oshi, Yoshihisa Tokumaru, Mariko Asaoka, Li Yan, Vikas Satyananda, Ryusei Matsuyama, Nobuhisa Matsuhashi, Manabu Futamura, Takashi Ishikawa, Kazuhiro Yoshida, Itaru Endo, Kazuaki Takabe

**Affiliations:** 1 Breast Surgery, Department of Surgical Oncology, Roswell Park Comprehensive Cancer Center, Elm & Carlton Streets, Buffalo, NY 14263 USA; 2 Department of Gastroenterological Surgery, Yokohama City University Graduate School of Medicine, Yokohama, 236-0004 Japan; 3 Department of Surgical Oncology, Graduate School of Medicine, Gifu University, 1-1 Yanagido, Gifu, 501-1194 Japan; 4 Department of Breast Oncology and Surgery, Tokyo Medical University, 6-7-1 Nishishinjuku, Shinjuku, Tokyo, 160-8402 Japan; 5 Department of Biostatistics and Bioinformatics, Roswell Park Comprehensive Cancer Center, Buffalo, NY 14263 USA; 6 Department of Surgical Oncology, Roswell Park Comprehensive Cancer Center, Buffalo, NY 14263 USA; 7 Department of Surgery, University At Buffalo Jacobs School of Medicine and Biomedical Sciences, The State University of New York, Buffalo, NY 14263 USA; 8 Department of Surgery, Niigata University Graduate School of Medical and Dental Sciences, Niigata, 951-8510 Japan; 9 Department of Breast Surgery, Fukushima Medical University School of Medicine, Fukushima, 960-1295 Japan

**Keywords:** Cancer microenvironment, Breast cancer

## Abstract

Tumor associated macrophages (TAMs) play a critical role in biology of various cancers, including breast cancer. In the current study, we defined “M1” macrophage and “M1”/“M2” ratio by transcriptomic signatures using xCell. We investigated the association between high level of “M1” macrophage or “M1”/“M2” ratio and the tumor immune microenvironment by analyzing the transcriptome of publicly available cohorts, TCGA and METABRIC. We found that “M1” high tumors were not associated with prolonged survival compared with “M1” low tumors, or with the response to neoadjuvant chemotherapy. “M1” high tumors were associated with clinically aggressive features and “M1” high tumors enriched the cell proliferation and cell cycle related gene sets in GSEA. At the same time, “M1” high tumors were associated with high immune activity and favorable tumor immune microenvironment, as well as high expression of immune check point molecules. Strikingly, all these results were mirrored in “M1”/“M2” ratio high tumors. In conclusion, transcriptomically defined “M1” or “M1”/“M2” high tumors were associated with aggressive cancer biology and favorable tumor immune microenvironment but not with survival benefit, which resembled only part of their conventional clinical characteristics.

## Introduction

Tumor immune microenvironment (TIME) plays critical role by contributing to the progression of various types of cancers, including breast cancer^1,2^. TIME consists of various types of immune cells^3^. Tumor associated macrophages (TAMs) are one of the major types of tumor infiltrating innate immune cells, playing a critical role in inflammation, which is well established to promote tumor growth and tumor progression^4,5^.

TAMs are known to polarize to either M1, which represents anti-tumor activity, or M2, which leads to tumor promotion^6^. Accumulating data from numerous studies demonstrated that TAMs are extremely plastic and heterogenous, and its polarization represents a continuum^6–8^. Thus, polarized TAMs are recently described as M1-like or M2-like macrophages^9^, rather than M1 or M2 macrophage, which are considered over-simplified^6,10,11^. May be due to its extreme plasticity, even the association between TAMs and patient prognosis i.e., M2-like macrophages associate with unfavorable survival and M1-like macrophages associate with favorable outcomes are currently questioned^12–14^. Other than survival, infiltration by M1 was reported to be associated with aggressive clinical features such as high Nottingham pathological grade and increased cell proliferation marker, Ki-67^15^. The balance between these two polarized macrophages is important because previous studies demonstrated that TAMs are predominantly M2-like macrophages and they are associated with cancer progression^16–18^.

Our group has been utilizing computational biological analyses on transcriptome of the bulk tumor to elucidate the clinical relevance of TIME^19–26^. The gold standard to analyze TIME is flow cytometry of fresh sample and/or immunohistochemistry of fixed slides. Although there is no doubt of usefulness of these technologies in basic research setting, their utility in large sample size of clinical patients can be challenging given the access to samples, costs and labor involved. To overcome this difficulty, we defined TAMs as “M1” and “M2” macrophages following the transcriptomic marker defined in the computational algorithm, xCell, developed by Aran et al.^27^. We defined “M1” macrophage and “M2” macrophage by utilizing the expression pattern of 188 and 159 genes respectively, as reported by Aran et al.^27^ (Supplementary Table S1, Supplementary Table S2). We analyzed the transcriptome of large breast cancer patient cohorts including The Cancer Genome Atlas (TCGA) and Molecular Taxonomy of Breast Cancer International Consortium (METABRIC).

We hypothesize that breast cancer with high level of “M1” macrophage and “M1”/“M2” ratio, both of which are defined by transcriptomic signatures using xCell, are associated with high infiltration of anti-cancer immune cells, and with positive clinical outcomes such as survival and response to neoadjuvant chemotherapy, which do not resemble their conventional clinical characteristics.

## Material and methods

### Data acquisition

Total of 1065 patients’ clinicopathological and gene expression data of The Cancer Genome Atlas (TCGA) Pan-Cancer study (TCGA PanCancer Atlas) was obtained through cBioPortal as previously described^26,28,29^. The data of Molecular Taxonomy of Breast Cancer International Consortium (METABRIC) database was obtained from cBioPortal to perform the validation analysis as previously described^22,30–32^. METABRIC cohort contained data for total of 1903 patients. Gene Expression Omnibus (GEO) data sets, GSE25066 and GSE22358, GSE20194, and GSE32646 were used for the analysis of the response to neoadjuvant chemotherapy^33–36^. GSE25066 contained 467 patients who received taxane-anthracycline chemotherapy. GSE22358 included 117 patients who received Docetaxel-Capecitabine with or without Trastuzumab. GSE20194 included 197 patients who received paclitaxel, 5-fluorouracil, cyclophosphamide and doxorubicin. GSE32646 contained 81patients who underwent 5-fluorouracil, epirubicin, cyclophosphamide and paclitaxel. We utilized these cohorts to assess the association between “M1” or “M1”/“M2” levels and the response rate to neoadjuvant chemotherapy (NAC). We divided “M1” as well as “M1”/“M2” into high and low group using median cutoff. The pathological complete response (pCR) rate, the percentage of patients that achieved disappearance of cancer cells confirmed pathologically, were calculated and those were used to compare the difference between “M1” or “M1”/“M2” high and low groups.

Given that all the cohorts used in this study, TCGA, METABRIC and GEO data sets, are de-identified publicly available database, Institutional Review Board was waived.

### Gene set enrichment analysis (GSEA)

Gene set enrichment analysis (GSEA) was performed using the publicly available software provided by Broad Institute (https://software.broadinstitute.org/gsea/index.jsp) as previously described^25,37–40^. Hallmark gene sets were used for this study. The statistical significance of GSEA was determined using false discovery rate (FDR) of 0.25 throughout the study as recommended by Broad Institute, the developer of GSEA.

### Immune cell composition and scores related with immune activity

Tumor associated macrophages, “M1” macrophages and “M2” macrophages, were defined utilizing 188 and 159 genes respectively following the definition used in xCell, a computational algorithm published in the journal, Genome Biology in 2017 by Aran et al.^27^. xCell was used to analyze all the other immune cell composition as well. The data of Leukocyte Fraction, Lymphocyte Infiltration Signature Score, IFN-gamma Response, TGF-beta Response, and TIL Regulation were adjusted with the previously reported study by Thorsson et al.^41^. Cytolytic activity (CYT) was calculated using the geometric mean of granzyme A and Perforin 1 expression values as described previously^22,31,42,43^.

### Statistical analysis

Statistical analysis was performed using R software (https://www.r-project.org/). Kaplan–Meier survival analysis was performed with greyzoneSurv packages in R for the survival analysis. One-way ANOVA or Fisher’s exact test were used to determine the significance of differences for groups. Fisher’s exact test was used comparing high and low “M1” or “M1”/“M2” ratio. Other box plots were analyzed with one-way ANOVA. A two-sided *p* value < 0.05 was considered statistically significant. All boxplots are of Tukey type, and the boxes depict medians and inter-quartile ranges.

## Results

### “M1” high tumors or “M1”/“M2” high tumors were not associated with favorable survival outcome compared to “M1” low tumors or “M1”/“M2” low tumors

First, we investigated the correlation between previously reported markers for M1 or M2 and our defined “M1” macrophage or “M2” macrophage. Previously, CD32, CD64, CD68, CD80, and CD86 were reported as the makers for M1 macrophages 44–46, and CD163, CD204, and CD206 for M2 macrophages^44–46^, and CD163, CD204, and CD206 for M2 macrophages^46,47^. For “M1” macrophage, all the gene investigated, CD32, CD64, CD68, CD80, and CD86, were strongly correlated with “M1” macrophage in TCGA cohort (Supplementary Fig. S1A). This was validated with another large cohort, METABRIC. On the other hand, “M2” macrophage demonstrated weak to moderate correlation with CD163 and CD204 and no correlation with CD206 (Supplementary Fig. S1B) in both TCGA and METABRIC cohorts.

**Figure 1:**
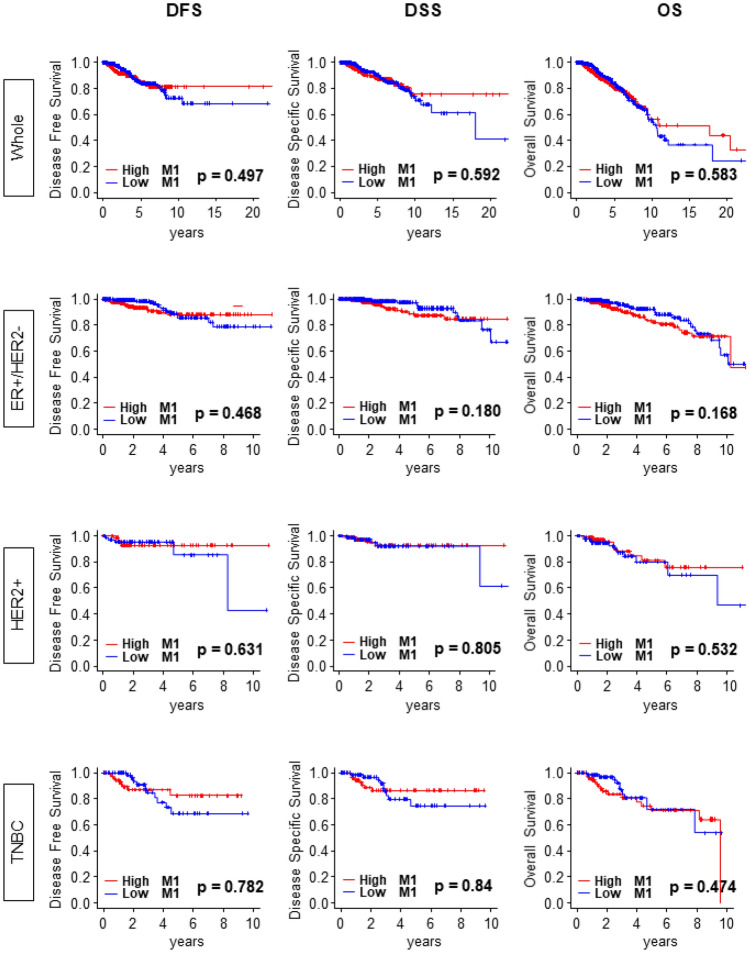
Kaplan Meier survival analysis of DFS, DSS, and OS did not demonstrate the survival benefit of “M1” high tumors over “M1” low tumors. DFS (Whole, n = 924, High = 466, low = 458, Hazard ratio (HR) 1.16 (95% Confidence interval [CI] 0.76–1.78); ER+/HER2−, n = 506, High = 253, Low = 253, HR 1.29 (CI 0.64–2.59); HER2+, n = 151, High = 81 Low = 70, HR 0.74 (CI 0.21–2.56); TNBC, n = 144, High = 73, Low = 71, HR 0.88 (CI 0.36–2.17). DSS (Whole, n = 1045, High = 526, low = 519, HR 1.13 (CI 0.73–1.74); ER+/HER2−, n = 572, High = 287, Low = 285, HR 1.67 (CI 0.78–3.56); HER2+, n = 173, High = 87, Low = 86, HR 0.85 (CI 0.22–3.21); TNBC, n = 153, High = 76, Low = 77, HR 0.91 (CI 0.35–2.35). OS (Whole, n = 1064, High = 532, Low = 532, HR 1.10 (CI 0.79–1.51); ER+/HER2−, n = 578, High = 289, Low = 289, HR 1.45 (CI 0.85–2.48); HER2+, n = 175, High = 88, Low = 87, HR 0.75 (CI 0.31–1.83); TNBC, n = 159, High = 80, Low = 79, HR 1.33 (CI 0.61–2.88). M1, “M1” macrophage; DFS, disease free survival; DSS, disease specific survival; OS, overall survival.

Based on the previous reports that M1 shifts the tumor immune microenvironment toward anti-cancer environment^1^, we expected that “M1” high tumors associate with favorable survival outcome. Surprisingly, “M1” high tumors did not demonstrate the survival benefit over “M1” low groups in the entire cohort (Whole) or in any of the subtypes of the TCGA breast cancer cohort. There was no difference in disease-free survival (DFS), disease specific survival (DSS), and overall survival (OS) and in Whole cohort (Fig. 1; *p* = 0.497, *p* = 0.592 and *p* = 0.583, respectively). This result was consistent with all the subtypes, estrogen receptor positive/human epidermal growth factor receptor 2 negative (ER+/HER2−), HER2 positive (HER2+) and triple negative breast cancer (TNBC) (Fig. 1). Lack of association between “M1” levels and survival were strikingly similar in “M1”/“M2” ratio as well. There was no significant survival difference between high “M1”/“M2” ratio tumors compared with low “M1”/“M2” ratio tumors except DFS in TNBC (Supplementary Fig. S2; *p* = 0.015).

### High expression of “M1” as well as high ratio of “M1”/“M2” was not associated with pathological complete response (pCR) to neoadjuvant chemotherapy (NAC)

**Figure 2:**
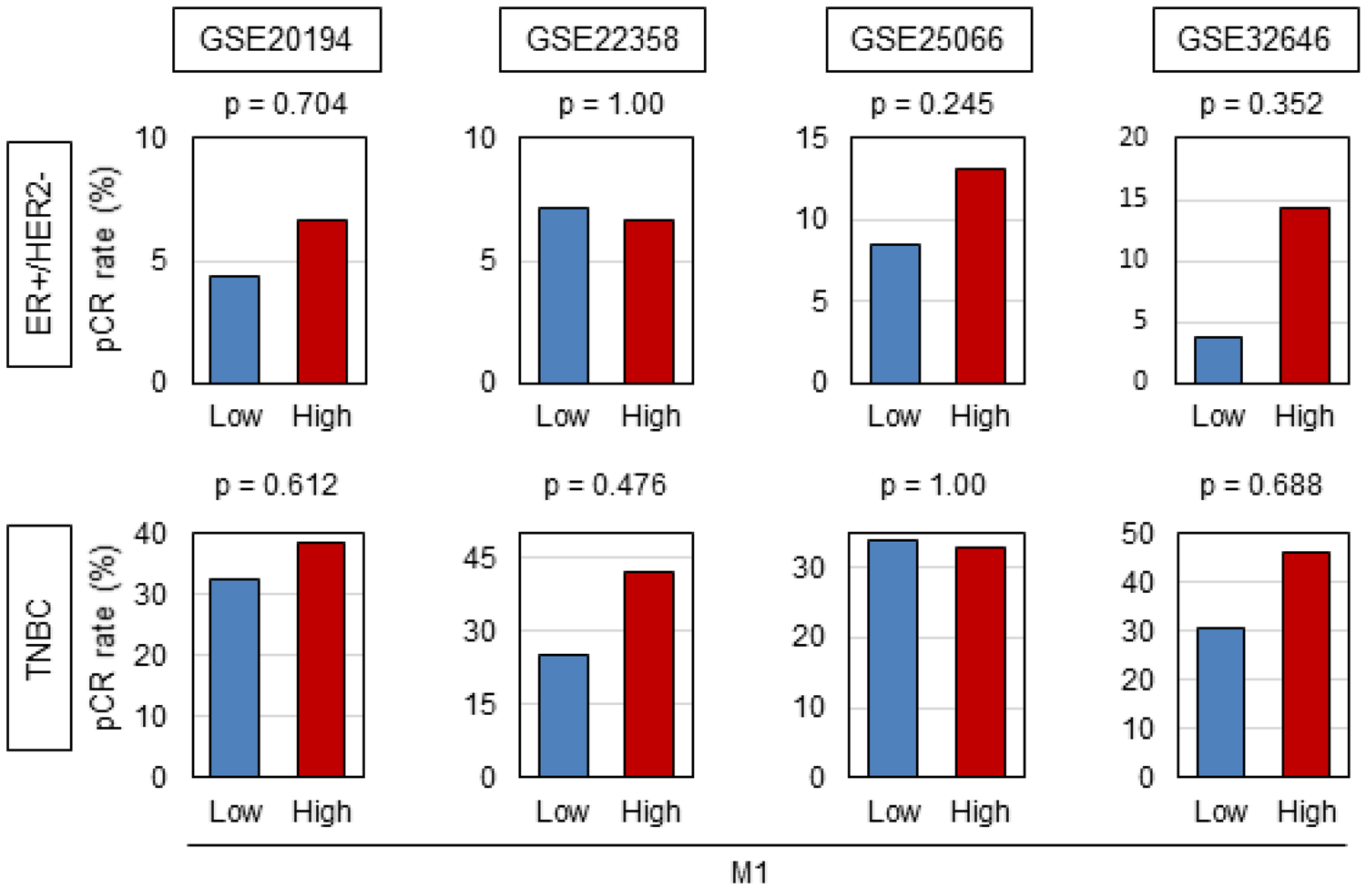
“M1” high tumors did not demonstrate the association with response to neoadjuvant chemotherapy. The association between expression of “M1” and pCR rate. GSE20194 (ER+/HER2−, n = 129, High = 60, Low = 69; TNBC, n = 68, High = 34, Low = 34). GSE22358 (ER+/HER2−, n = 67, High = 34, Low = 33; TNBC, n = 50, High = 25, Low = 25). GSE25066 (ER+/HER2−, n = 289, High = 145, Low = 144; TNBC, n = 178, High = 90, Low = 88). GSE32646 (ER+/HER2−, n = 55, High = 28, Low = 27; TNBC, n = 26, High = 13, Low = 13). TNBC, triple negative breast cancer.

Previous report demonstrated that high infiltration level of TAMs is associated with higher response rate to NAC^48^. Thus, we expected that “M1” macrophage is associated with increased pCR to NAC. Four NAC cohorts, GSE20194 (n = 197) that underwent paclitaxel, 5-fluorouracil, cyclophosphamide and doxorubicin, GSE22358 (n = 117) that underwent Docetaxel–Capecitabine ± Trastuzumab, GSE25066 (n = 467) that underwent taxane-anthracycline chemotherapy and GSE32646 (n = 81) that underwent 5-fluorouracil, epirubicin, cyclophosphamide, and paclitaxel were analyzed^33–36^. “M1” high tumors were not associated with pCR rate in any of the subtypes. These results indicated that high “M1” tumors were not associated with pCR to NAC (Fig. 2). Also, there was no association between “M1”/“M2” high tumors and pCR rate of NAC (Supplementary Fig. S3).

### Infiltration of “M1” macrophage and “M1/M2” high tumors were associated with subtypes and Nottingham pathological grade, but not with cancer staging

**Figure 3:**
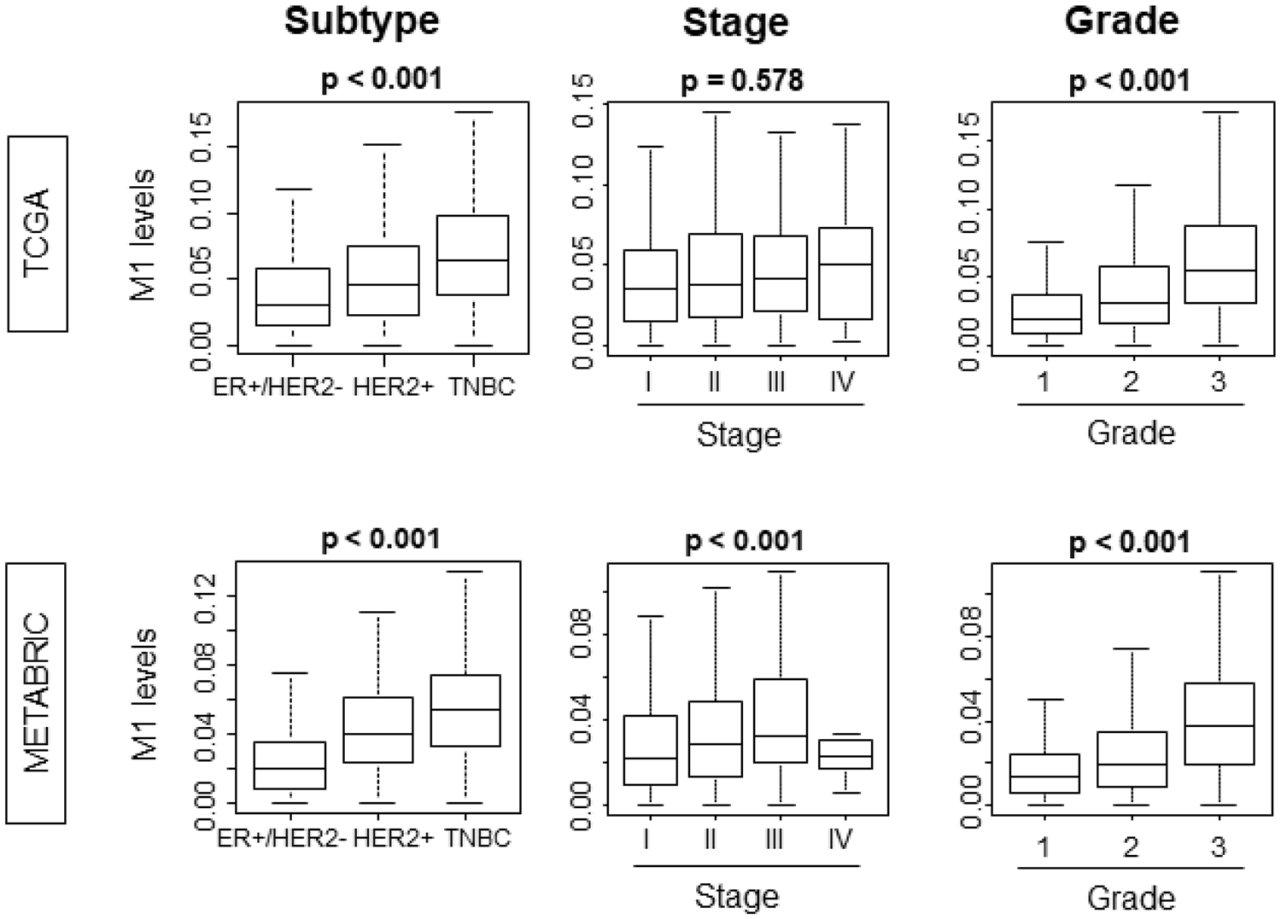
The expression levels of “M1” was different between the subtypes and higher number of infiltrating “M1” was associated with more advanced grade. TNBC, triple negative breast cancer.

Next, we explored the association between infiltration of “M1” and clinical parameters of aggressive tumors, such as cancer staging, subtypes, and Nottingham pathological grade. Among the subtypes, ER+/HER2−, which is the least aggressive, had the lowest level of infiltrating “M1”, whereas triple negative (TN), the most aggressive subtype, demonstrated the highest level (Fig. 3; *p* < 0.001). This result was validated by another independent cohort METABRIC, which completely echoed the result (Fig. 3; *p* < 0.001). “M1” levels trend to increase with clinical staging. METABRIC demonstrated statistically significant difference especially from Stage I to Stage III, however, this was not the case in TCGA, which may be from its smaller sample size. Infiltration of TAMs was reported to be associated with Nottingham pathological grade, which is a pathological determination of cancer aggressiveness^49^. We found that high “M1” level was associated with advanced grade (Fig. 3; *p* < 0.001). This result was mirrored in validation METABRIC cohort (Fig. 3; *p* < 0.001).

### “M1” high tumors and “M1”/“M2” high tumors enriched cell cycle and cell proliferation related gene sets and were associated with high Ki67 expression

**Figure 4:**
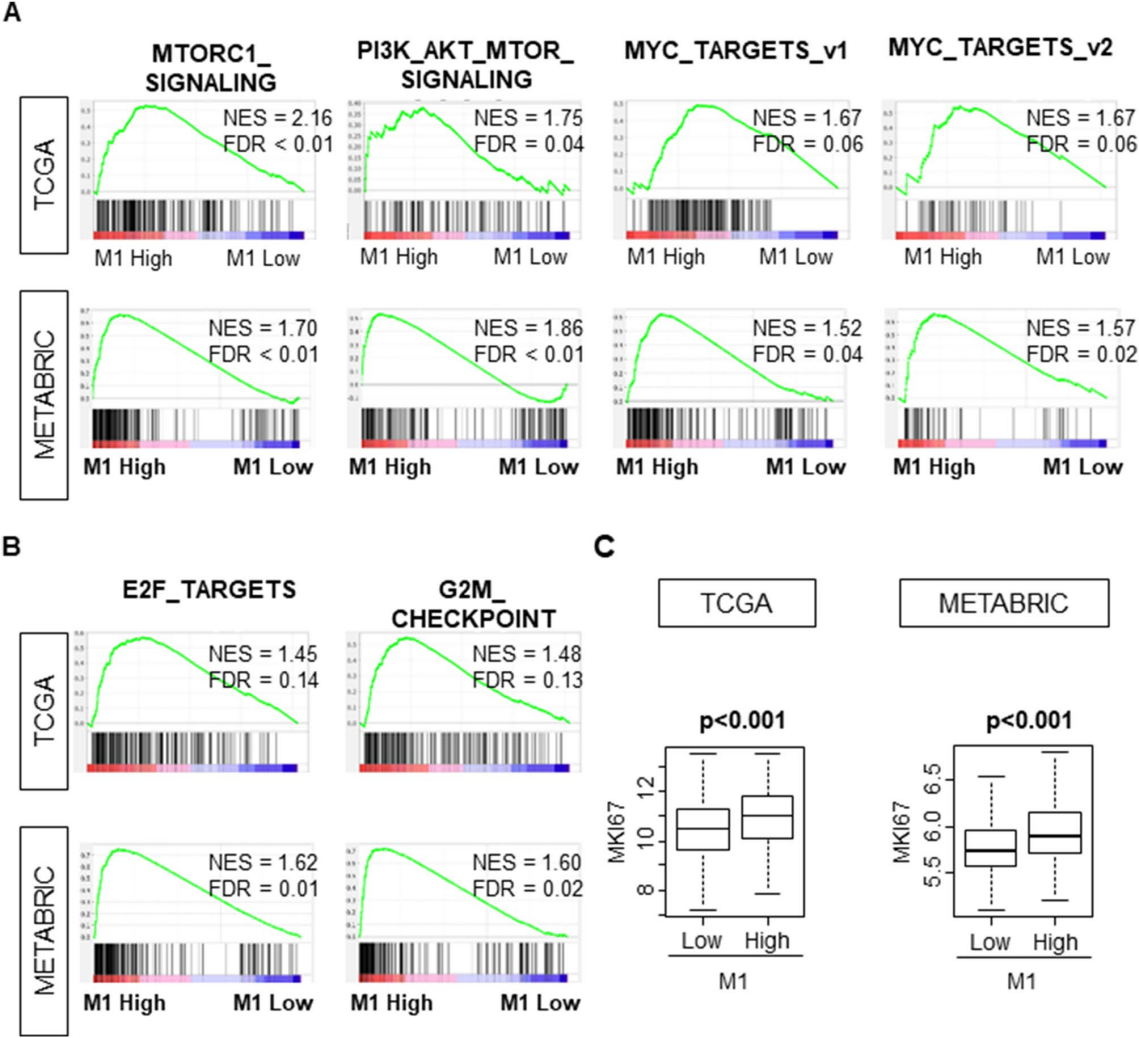
“M1” high tumors were associated with highly proliferative features. (**A**) “M1” high tumors were associated with the gene sets related to cell proliferation. (**B**) “M1” high tumors were associated with the gene sets related to cell proliferation. (**C**) “M1” high tumors were associated with Ki-67 transcriptome analysis in both TCGA and METABRIC cohort. TCGA (n = 1065, High = 533, Low = 532). METABRIC (n = 1903, High = 953, Low = 950). The statistical significance of GSEA was determined using false discovery rate (FDR) of 0.25.

Given that Nottingham pathological grade was associated with high infiltration of M1^15^, we hypothesized that “M1” high tumors are associated with highly proliferative cancer. Gene Set Enrichment Analysis (GSEA) using TCGA cohort as testing cohort demonstrated that high “M1” level significantly enriched gene sets related to cell proliferation, such as, MTORC1_SIGNALING, PI3K_AKT_MTOR_SIGNALING, MYC_TARGETS_v1, MYC_ TARGETS_v2 (Fig. 4A, ; NES = 2.16, FDR < 0.01; NES = 1.75, FDR = 0.04; NES = 1.67 , FDR = 0.06; NES = 1.67 , FDR = 0.06 respectively). These results were completely mirrored in validation METABRIC cohort (Fig. 4A; NES = 1.70, FDR < 0.01; NES = 1.86, FDR < 0.01; NES = 1.52, FDR = 0.04; NES = 1.57, FDR = 0.02, respectively). Furthermore, high “M1” level significantly enriched the gene sets related with cell cycle, such as E2F TARGETS and G2M_CHECKPOINT, were also enriched with high “M1” tumors (Fig. 4B; NES = 1.45, FDR = 0.14; NES = 1.48, FDR = 0.13, respectively) in testing TCGA cohort, which was also completely mirrored in validation METABRIC cohort (Fig. 4B; NES = 1.62, FDR = 0.01; NES = 1.60, FDR = 0.02, respectively).

This notion was further confirmed by transcriptome analysis of Ki-67, which is one of the most commonly used markers for cell proliferation. High “M1” tumors were associated with significantly higher Ki-67 expression (Fig. 4C; *p* < 0.001). This result was validated with METABRIC (Fig. 4C; *p* < 0.001). Interestingly, “M1”/“M2” high tumors enriched the gene sets related to cell proliferation in only METABRIC cohort whereas ‘M1”/“M2” high tumors were associated with higher Ki-67 expression in both TCGA and METABRIC cohorts (Supplementary Fig. S4).

### “M1” high tumors and “M1”/“M2” high tumors enriched immune activity related gene sets and were associated with both favorable and unfavorable immune activity

**Figure 5:**
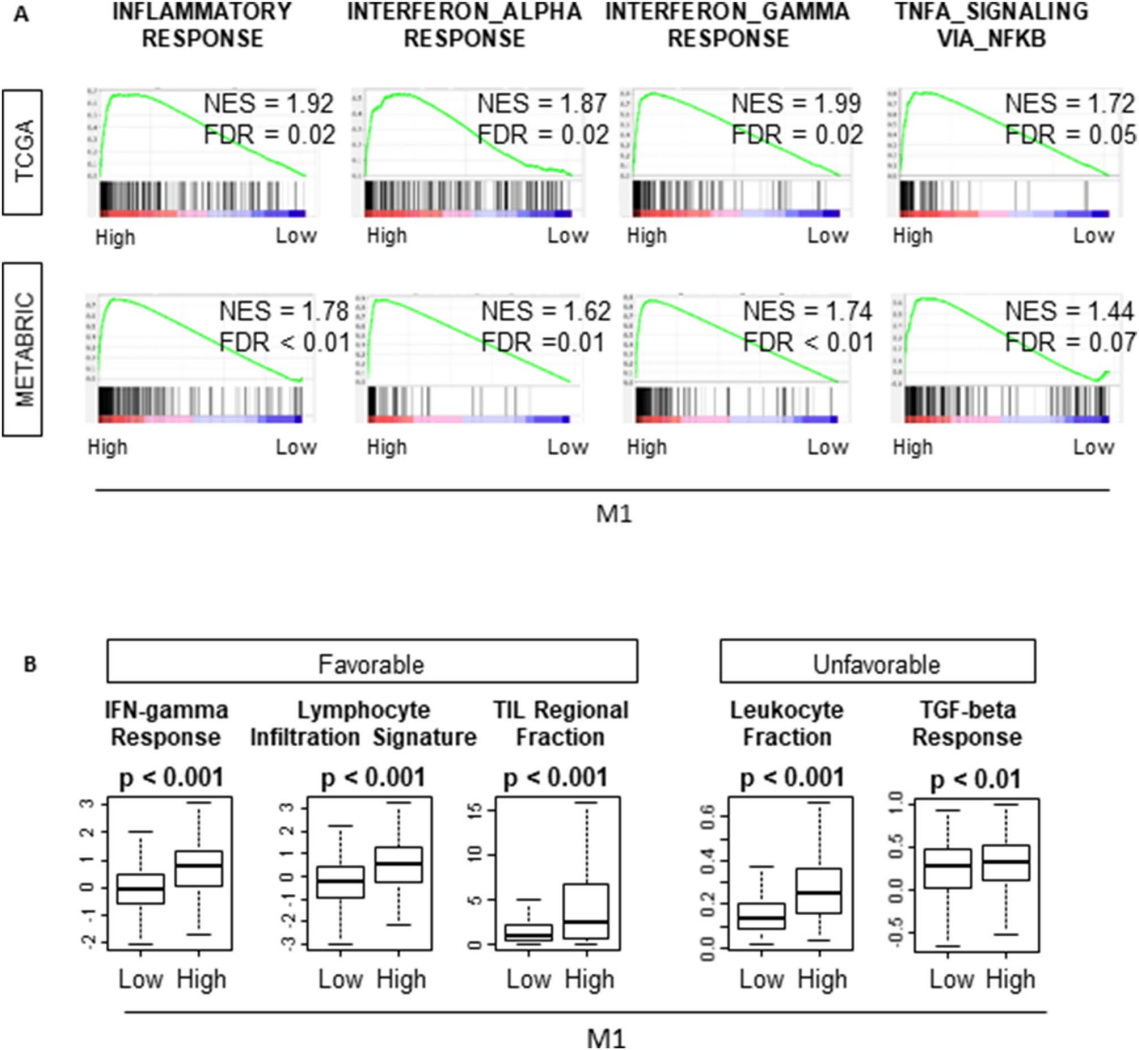
“M1” high tumors enriched the immune associated gene sets and demonstrated higher immune activity related scores. (**A**) “M1” high tumors enriched the gene sets related immune activity. (**B**) High infiltration of “M1” was associated with higher score of both favorable and unfavorable immune activity related scores. TCGA (n = 1065, High = 533, Low = 532). METABRIC (n = 1903, High = 953, Low = 950). The statistical significance of GSEA was determined using false discovery rate (FDR) of 0.25.

Taken together that “M1” high tumors were significantly associated with highly proliferative aggressive cancer but did not associate with worse survival, we hypothesized that “M1” high tumors were associated with high immune activity that counterbalance the cancer aggressiveness. GSEA demonstrated that “M1” high tumors enriched the gene sets related with immune activity, such as INFLAMATORY_RESPONSE, INTERFERON_ALPHA_RESPONSE, INTERFERON_GAMMMA_RESPONSE, TNFA_SIGNALING (Fig. 5A). These results were completely echoed in the validation cohort, METABRIC (Fig. 5A). However, “M1”/“M2” high tumors enriched INFLAMATORY_RESPONSE, INTERFERON_ALPHA_RESPONSE, and INTERFERON_GAMMMA_RESPONSE in METABRIC cohort only (Supplementary Fig. S5).

As expected, “M1” high tumors demonstrated significantly higher favorable immune activity, such as, Lymphocyte Infiltration Signature Score, TIL Regional Fraction Score and IFN-gamma Response in TCGA cohort (Fig. 5B). Somewhat unexpected was that “M1” high tumors also demonstrated elevation of unfavorable immune activity such as Leukocyte Fraction and TGF-beta Response at the same time (Fig. 5B). These results were similar with “M1”/“M2” high tumors. “M1”/“M2” high tumors demonstrated significantly higher favorable activity scores, as well as unfavorable immune activity scores (Supplementary Fig. S5). These analyses were only available in TCGA cohort^41^, thus, there is no data on METABRIC.

### “M1” high tumors and “M1”/“M2” high tumors were associated with both favorable and unfavorable tumor immune microenvironment

**Figure 6:**
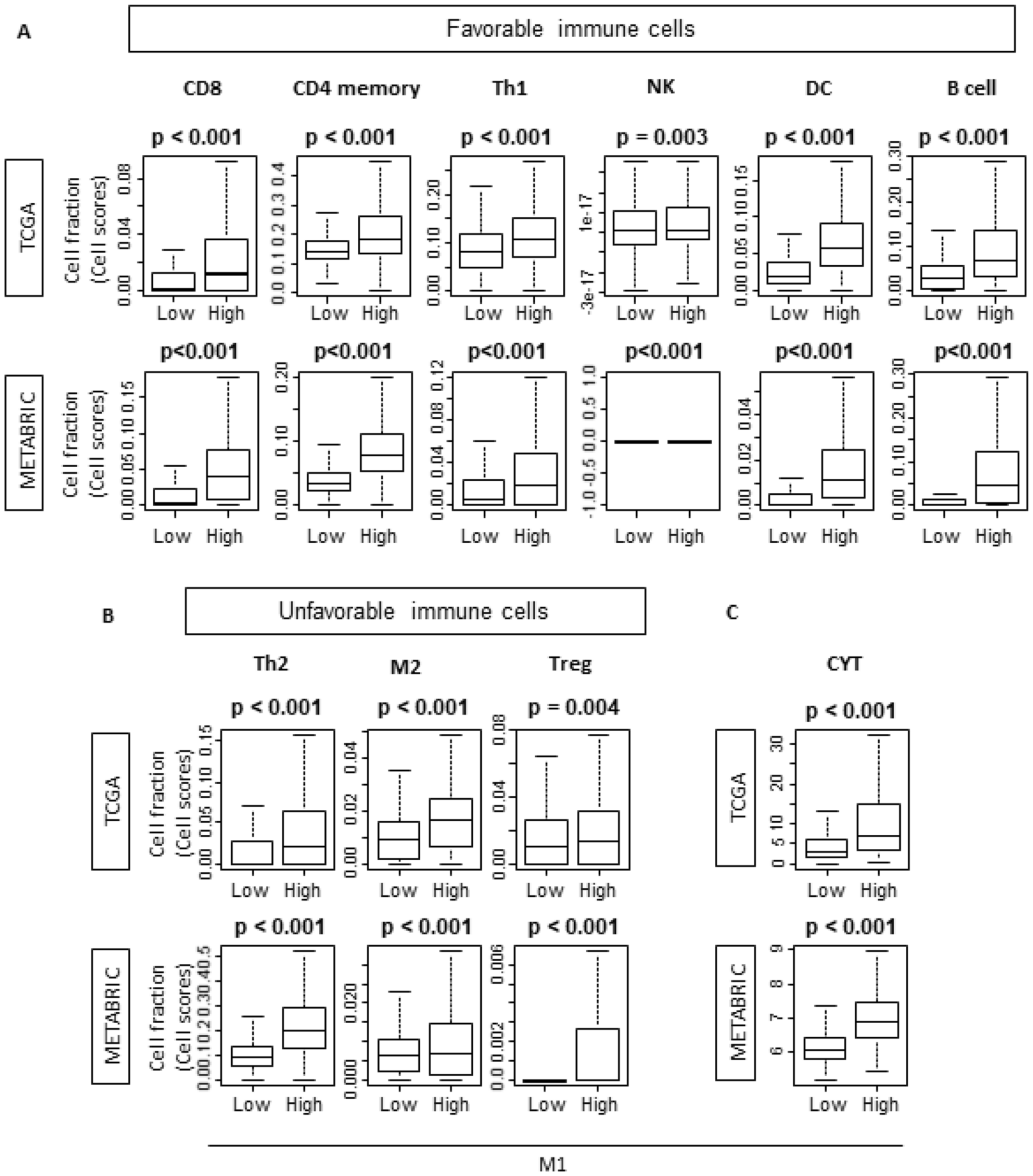
“M1” high tumors demonstrated the infiltration of both favorable and unfavorable immune cells in TCGA and METABRIC cohort. T-helper 1/2; DC, Dendritic Cell; Treg, regulatory T cell; CYT, cytolytic activity score. TCGA (n = 1065, High = 533, Low = 532). METABRIC (n = 1903, High = 953, Low = 950).

As “M1” high tumors were associated with higher immune activity, we further explored the association of “M1” and infiltrating immune cells in tumor immune microenvironment using xCell, the computational algorithm to analyze the immune cell composition^27^. “M1” high group had significantly higher rate of anti-cancer immune cell infiltration, such as CD8+ cells, CD4 memory cells, T-helper 1 (Th1) cells, natural killer (NK) cells , dendritic cells (DC) and B cells in TCGA cohort (Fig. 6A; *p* < 0.001, *p* < 0.001, *p* < 0.001, *p* < 0.01, *p* < 0.001, and *p* < 0.001 respectively). These results were mirrored by validation METABRIC cohort, although NK cells were detected only in 33 cases in “M1” high group. In agreement with the immune activity scores, pro-cancer immune cells, Th2 cells, “M2” cells and regulatory T cells (Tregs) were also highly infiltrated in “M1” high tumors in testing TCGA cohort (Fig. 6B; *p* < 0.001, *p* < 0.001 and *p* < 0.01 respectively), which were mirrored in validation METABRIC cohort (Fig. 6B; *p* < 0.001, *p* < 0.001 and *p* < 0.001 respectively). Since cytolytic activity score (CYT) is significantly high in high “M1” tumors, overall, high “M1” tumors may associate with favorable tumor immune microenvironment (Fig. 6C; *p* < 0.001 and *p* < 0.001 respectively). These trends were also demonstrated with “M1”/“M2” high tumors (Supplementary Fig. S6).

### “M1” high tumors and “M1”/“M2” high tumors were associated with higher T cells exhaustion marker

**Figure 7:**
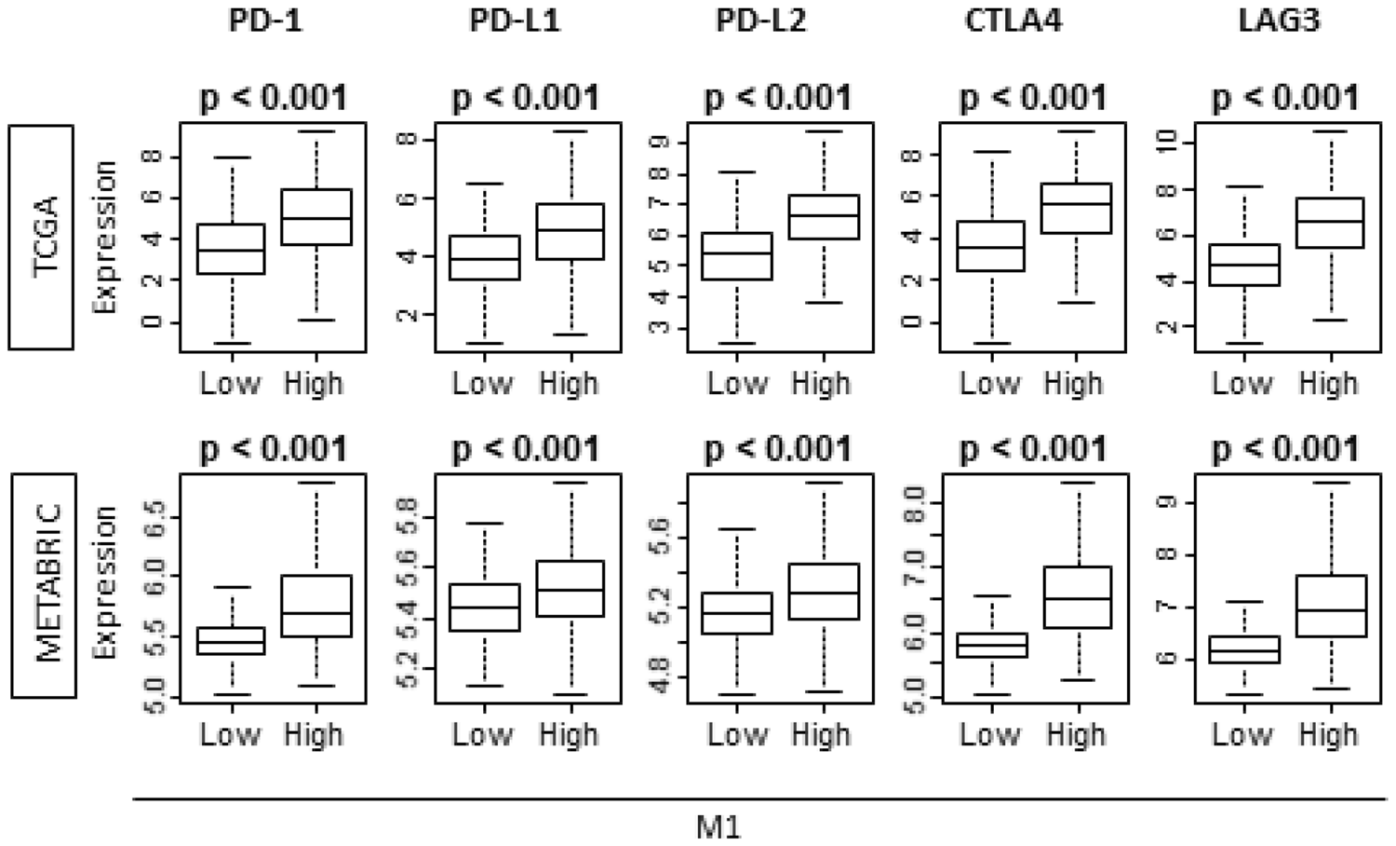
“M1” high tumors demonstrated the association with T cell exhaustion markers in both TCGA and METABRIC cohort. PD-1, programmed death-1; PD-L1/2, (programmed death ligand 1/2); CTLA4, cytotoxic T-lymphocyte-associated protein 4; LAG3, lymphocyte activation gene 3. TCGA (n = 1065, High = 533, Low = 532). METABRIC (n = 1903, High = 953, Low = 950).

Because “M1” high tumors possess higher immune activity and attract more immune cells, we speculated that T cell exhaustion markers will be upregulated within those tumors. We found that all exhaustion markers, PD1, PD-L1, PD-L2, CTLA4, and LAG3, were significantly higher in “M1” high tumors in testing TCGA cohort (Fig. 7). These results were validated with validation METABRIC cohort (Fig. 7). Strikingly, these results were also echoed with “M1”/“M2” high tumors in both TCGA and METABRIC cohorts (Supplementary Fig. S7).

## Discussion

Despite previous reports that high infiltration of M1-like macrophage was associated with better survival outcome, we found that transcriptomically defined “M1” high tumors were not associated with favorable survival outcome with regards to OS, DSS, and DFS. We demonstrated that the infiltration of “M1” is not predictive for pCR to neoadjuvant chemotherapy (NAC). High “M1” tumors were associated with aggressive features, such as high Nottingham pathological grade and Ki-67. We found that high “M1” tumors enriched the gene sets related to cell proliferation and cell cycle with GSEA. The tumors with high “M1” infiltration enriched the immune related gene sets with GSEA and were associated with both favorable and unfavorable immune related scorings. High “M1” tumors were also associated with high infiltration of both favorable and unfavorable immune cells. Additionally, we found that high “M1” tumors possessed significantly higher cytolytic activity score, which may suggest overall favorable tumor immune microenvironment (TIME). Interestingly, “M1”/“M2” high tumors were not associated with favorable survival outcome in TCGA and were not predictive marker of response to NAC. “M1”/“M2” high tumors were associated with aggressive features. However, “M1”/“M2” high tumors enriched gene sets related to cell proliferation and cell cycles with GSEA in METABRIC cohort only. Furthermore, “M1”/“M2” high tumors were overall associated with favorable TIME.

Previously, polarized TAMs were recognized with oversimplified terms such as M1 macrophages and M2 macrophages based on the cell polarization concept of helper T cell type 1 and type 2^6,10,11^. However, with accumulating in vivo data from the studies of inflammatory diseases including cancer demonstrated that macrophage polarization is the representation of continuum of diverse functional state^6–8^. Therefore, currently the terms M1-like macrophages and M2-like macrophages are used to describe the phenotypes of polarized macrophages^9^. This is due to the extreme plasticity of macrophages in the tumor microenvironment. Polarization of TAMs are regulated by various interconnected pathways. For instance, the polarization to M1-like macrophages results with the predominant activation of STAT1 pathway. On the contrary, the activation of STAT3 and STAT6 pathways leads to the polarization to M2-like macrophages^6,50^.

M1-like macrophage demonstrate the polarized state which represents the anti-tumor activity whereas M2-like macrophage demonstrate the state which leads to tumor promotion^6^. M1-like macrophages release the cytokines and chemokines to induce inflammation which leads to the anti-tumor activity^6,51,52^. On the contrary, M2-like macrophages associate with angiogenesis, immune suppression, and tissue repair by interacting with the cytokines and chemokines which lead to tumor progression^6,51–53^. Ma et al. demonstrated that roughly 70% of TAMs infiltrated in non-small cell lung cancer are M2-like and the higher density of M1-like macrophages associated with better survival^16^. In the current study, we analyzed the clinical characteristics of transcriptomically defined macrophage “M1” as well as “M1”/“M2” ratio. The balance between these two polarized macrophages is important because previous studies demonstrated that TAMs are predominantly M2-like macrophages and they are associated with cancer progression. Recently, increase in M1-like/M2-like macrophage ratio in TIME, also known as “remodeling of the imbalance”, has been reported to associate with response to chemotherapy^16–18^. TAMs are not fixed with irreversible phenotype and M2-like macrophages can be converted and polarized into the anti-tumoral macrophage, M1-like macrophage, with change in TIME by intervention due to its extreme plasticity^17,54^. These facts led us to analyze the clinical relevance of “M1”/“M2” macrophages ratio.

The aim of the current study was to elucidate the clinical relevance of transcriptomically defined “M1” macrophage levels and “M1”/“M2” ratio in multiple large breast cancer patient cohorts. Because of the extreme plasticity of TAMs and its polarization is a spectrum, we utilized the oversimplified transcriptomic definition of “M1” and “M2” macrophages of Aran et al. Transcriptome analysis has emerged as the methodology to objectively evaluate the cells and this method allows us to analyze multiple large cohorts, such as TCGA and METABRIC. Our group has been publishing number of papers in which we used bioinformatic approach utilizing transcriptome data to assess the clinical relevance of genes of interest or the association of TIME in various types of cancers^19–26^. Computational algorithms allow us to estimate the cell composition of the immune cells, which enable us to understand more about TIME. Furthermore, we can determine the upregulated signaling of interest by performing GSEA. .

Previous reports demonstrated that high infiltration of M1 was associated with better survival with breast cancer patients^12^. To our surprise, “M1” high tumors did not demonstrate better prognostic outcome compared with “M1” low tumors. Given that M1 is well known to function as anti-cancer and “M1” high tumors were associated with more favorable TIME with current study, this result seems to be contradictory. This may be because “M1” high tumors demonstrated more aggressive clinical features in the current study. “M1” high tumors exhibit higher proliferation with a transcriptomic analysis of Ki-67 as well as GSEA. GSEA demonstrated that “M1” high tumors enriched the gene sets related to cell cycle and cell proliferation. Previously we reported that biological aggressive feature counterbalanced the anti-cancer TIME which resulted in an absence of significant difference in survival outcome in high mutation breast cancer^22^. In that study, high mutation tumors were associated with biological aggressive features, such as higher grade and cell proliferation, but were also associated with anti-cancer TIME at the same time. We speculate that the anti-tumor immune microenvironment shown as higher cytolytic activity associated with “M1” high tumor is offset by its association with highly proliferative cancer shown as enrichment of cell proliferation-related gene sets and high MKI67, which result in insignificant survival difference by “M1” macrophage levels. This notion agrees with our earlier study that showed that anti-cancer immunity and biologically aggressive phenotype counterbalance in breast cancer with high mutation rate^22^.

The result of the current study suggests that inflammation that recruits immune cells alone is not enough to suppress the tumor. We speculate that specificity of antigen presenting cells, such as macrophage, may need to be taken into consideration and probed further for development of an anti-tumor response. To this end, we plan to further investigate the specificity of other antigen presenting cells such as conventional dendritic cells and plasmacytoid dendritic cells utilizing in silico computational biological approach.

Despite the approval of immune check point inhibitors (ICIs) for triple negative breast cancer as the result of IMpassion130 trial was a breakthrough for the breast cancer community, the efficacy of ICIs is confined to a very limited population and the patient selection remains a major challenge^55,56^. Not only does our result demonstrate the upregulation of PD-L1 expression with “M1” high tumors, but it also demonstrates that other major T cell exhaustion markers, such as PD-1, CTLA4, and LAG3, were also upregulated. Given this result, we cannot help but speculate that “M1” high tumors could be the population who would respond to ICIs of any subtype of the breast cancer patients.

Our current study has some limitations. The biggest limitation of the current study is that we utilized xCell that defined “M1” and other immune cells by transcriptomic profiles alone, which may or may not grasp most of the M1-like macrophages, since polarization of TAM is a spectrum. Also, this is a retrospective study mainly using publicly available databases, TCGA and METABRIC. Although these databases provide us very informative clinical data and RNA sequencing data, they lack data regarding co-morbid conditions and therapeutic information. Further, it would be of interest to investigate the “M1” phenotype in normal breast tissue. At this point, analyses will not be statistically meaningful given the small sample size of normal breast tissue in TCGA and such dataset is awaited. This study does not contain in vitro and in vivo data. Therefore, further experiments are needed to assess the underlying mechanism of our findings in this study.

In conclusion, transcriptomically defined “M1” or “M1”/“M2” high tumors were associated with aggressive cancer biology and favorable tumor immune microenvironment but not with survival benefit, which are only part of their conventional clinical characteristics.

## Supplementary information

Supplementary Information.
