# Supplementary material for: M1 Macrophage and M1/M2 ratio defined by transcriptomic signatures resemble only part of their conventional clinical characteristics in breast cancer
Source: Sci Rep. 2020 Oct 6;10:16554. doi: 10.1038/s41598-020-73624-w (PMC7538579; doi:10.1038/s41598-020-73624-w)
Supplement: Supplementary file 1 — Supplementary Information. [file 41598_2020_73624_MOESM1_ESM.docx]

**M1 Macrophage and M1/M2 ratio defined by transcriptomic signatures resemble only part of their conventional clinical characteristics in breast cancer**

Masanori Oshi^1,2†^, Yoshihisa Tokumaru^1,3†^, Mariko Asaoka^1,4†^, Li Yan^5^, Vikas Satyananda^6^, Ryusei Matsuyama^2^, Nobuhisa Matsuhashi^3^, Manabu Futamura^3^, Takashi Ishikawa^4^, Kazuhiro Yoshida^3^, Itaru Endo^2^ and Kazuaki Takabe ^1,2,4,7-9^*

* Authors to whom correspondence should be addressed

^†^ These authors contributed equally

^1^ Breast Surgery, Department of Surgical Oncology, Roswell Park Comprehensive Cancer Center, Buffalo, NY 14263, USA

^2^ Department of Gastroenterological Surgery, Yokohama City University Graduate School of Medicine, Yokohama 236-0004, Japan.

^3^ Department of Surgical Oncology, Graduate School of Medicine, Gifu University, 1-1 Yanagido, Gifu 501-1194, Japan

^4^ Department of Breast Oncology and Surgery, Tokyo Medical University, 6-7-1 Nishishinjuku, Shinjuku, Tokyo, 160-8402, Japan.

^5^ Department of Biostatistics & Bioinformatics, Roswell Park Comprehensive Cancer Center, Buffalo, NY, 14263, USA

^6^ Department of Surgical Oncology, Roswell Park Comprehensive Cancer Center, Buffalo, NY 14263, USA

^7^ Department of Surgery, University at Buffalo Jacobs School of Medicine and Biomedical Sciences, The State University of New York, Buffalo, NY 14263, USA.

^8^ Department of Surgery, Niigata University Graduate School of Medical and Dental Sciences, Niigata 951-8510, Japan.

^9^ Department of Breast Surgery, Fukushima Medical University School of Medicine, Fukushima 960-1295, Japan

**
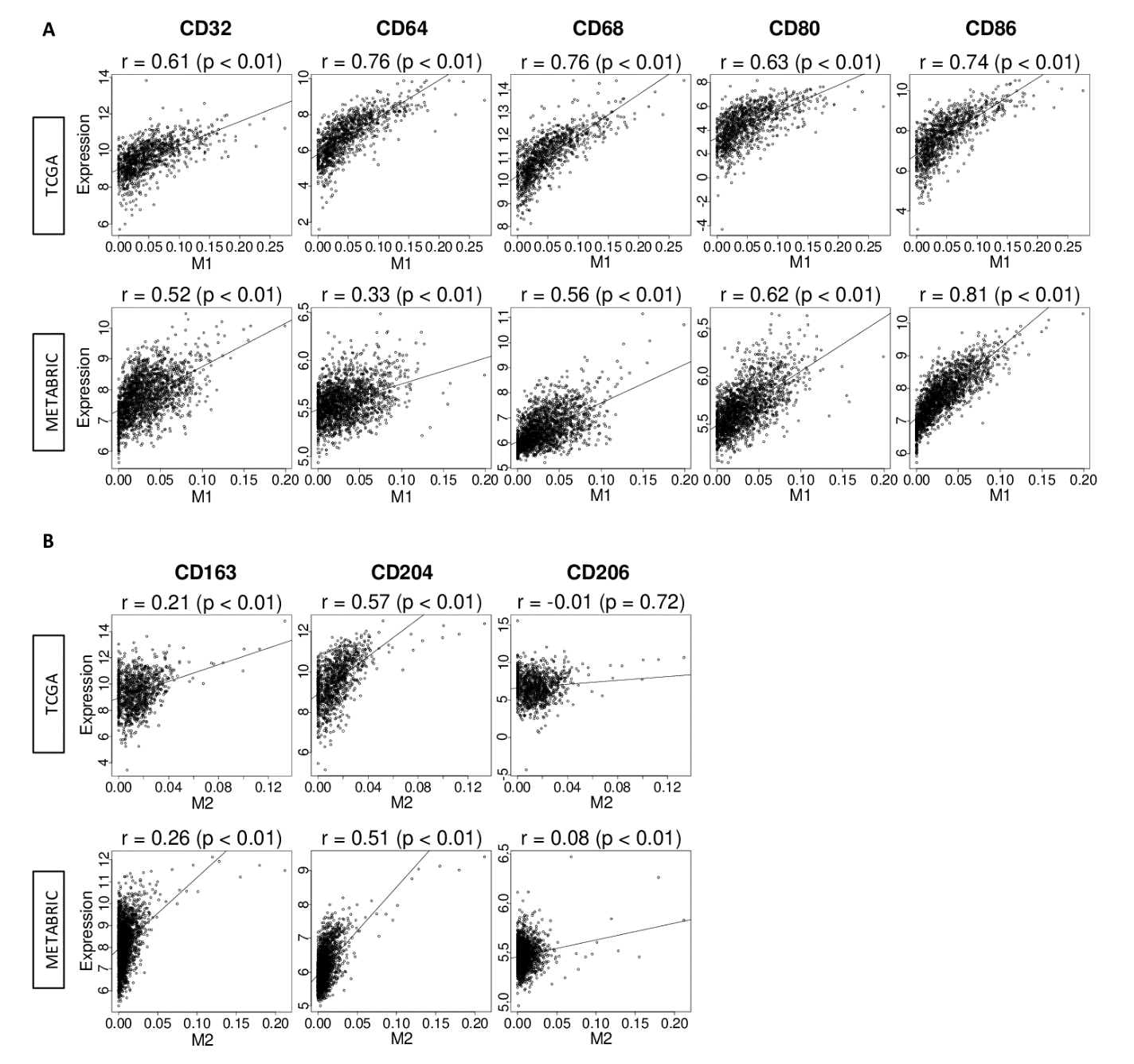
**

**Supplementary Figure S1.** (A) “M1” macrophage demonstrated the correlation between previously reported markers of M1 in both TCGA and METBRIC cohort. (B) ”M2” macrophage demonstrated correlation to some of the previously reported markers of M2 in both TCGA and METABRIC cohort.

**
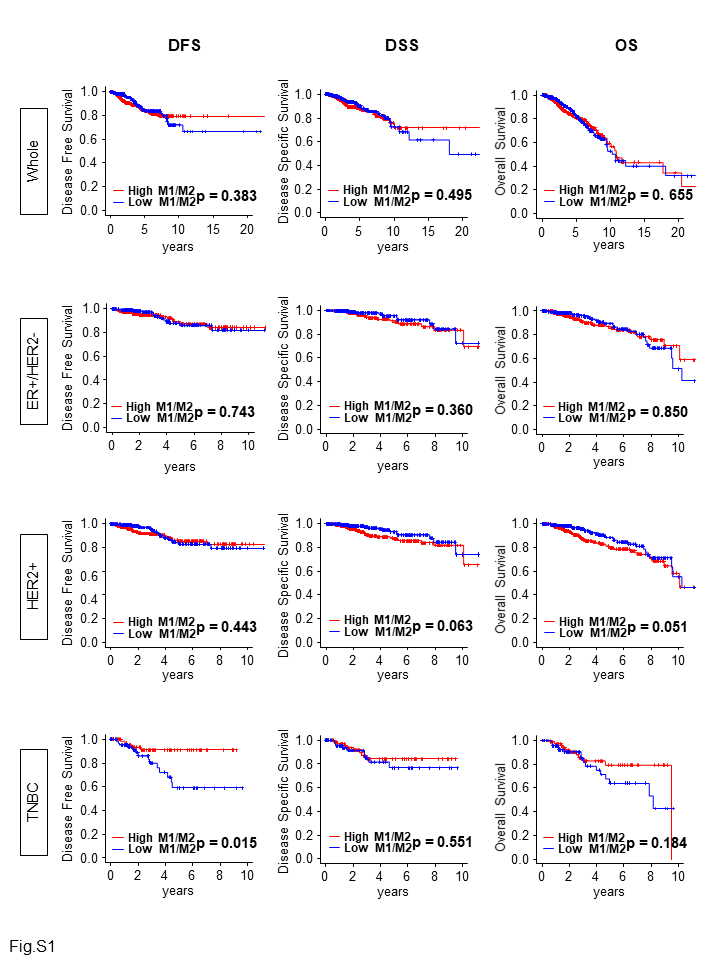
**

**Supplementary Figure S2.** Kaplan Meier survival analysis of DFS, DSS, and OS did not demonstrate the survival benefit of “M1”/”M2” high tumors over “M1”/”M2” low tumors except DFS of TNBC. DFS (Whole, n = 924, High = 468, Low = 456, Hazard ratio (HR) 1.21 (95% Confidence interval [CI]; 0.79-1.86); ER+/HER2-, n = 506, High = 252, Low = 254, HR 1.12 (CI; 0.56-2.25); HER2+, n = 132 , High = 68, Low = 64, HR 0.51 (CI; 0.12-2.15); TNBC, n = 144, High = 71, Low = 73, HR 0.30 (CI; 0.11-2.17)). DSS (Whole, n = 1045, High = 523, low = 522, HR 1.16 (CI; 0.75-1.80); ER+/HER2-, n = 572, High = 288, Low = 284, HR 1.42 (CI; 0.67-3.04); HER2+, n = 153, High = 76, Low = 77, HR 0.41 (CI; 0.11-1.53); TNBC, n = 153, High = 78, Low = 75, HR 0.75 (CI; 0.29-1.94)). OS (Whole, n = 1064, High = 532, low = 532, HR 1.08 (CI; 0.78-1.48); ER+/HER2-, n = 578, High = 289, Low = 289, HR 1.05 (CI; 0.62-1.79); HER2+, n = 156, High = 78, Low = 78, HR 0.42 (CI; 0.17-1.04); TNBC, n = 159, High = 80, Low = 79, HR 0.60 (CI; 0.28-1.29)). M1, “M1” macrophage; M2, “M2” macrophage; DFS, disease free survival; DSS, disease specific survival; OS, overall survival.

**
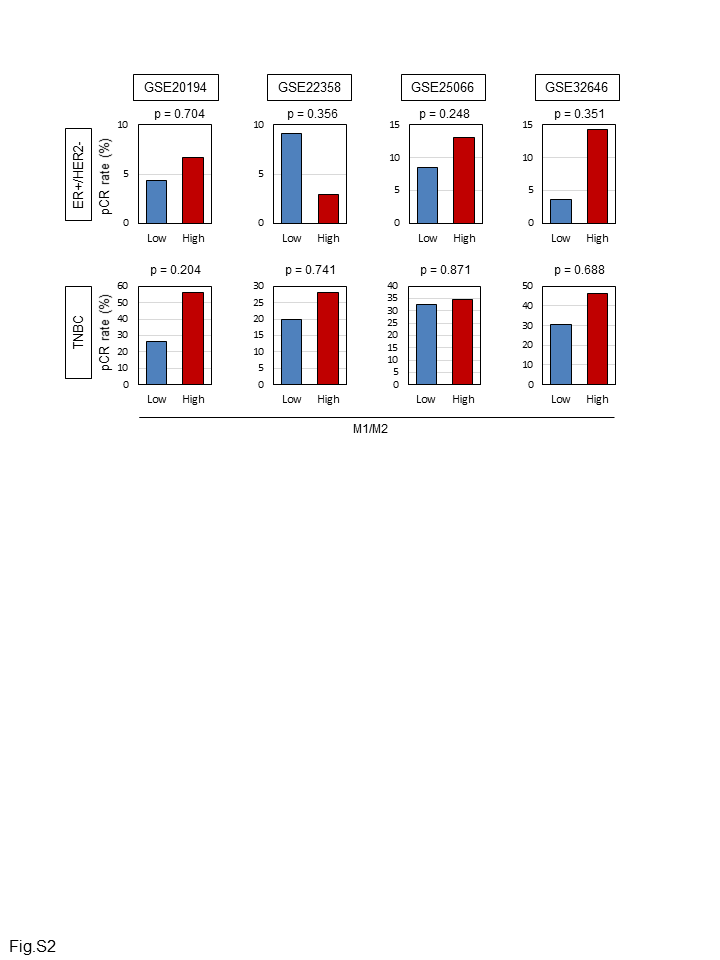
**

**Supplementary Figure S3.** “M1”/”M2” high tumors did not demonstrate the association with response to neoadjuvant chemotherapy. The association between expression of “M1”/”M2” and pCR rate. GSE20194 (ER+/HER2-, n = 129, High = 60, Low = 69; TNBC, n = 68, High = 34, Low = 34). GSE22358 (ER+/HER2-, n = 67, High = 34, Low = 33; TNBC, n = 50, High = 25, Low = 25). GSE25066 (ER+/HER2-, n = 289, High = 145, Low = 144; TNBC, n = 178, High = 90, Low = 88). GSE32646 (ER+/HER2-, n = 55, High = 28, Low = 27; TNBC, n = 26, High = 13, Low = 13). TNBC, triple negative breast cancer.


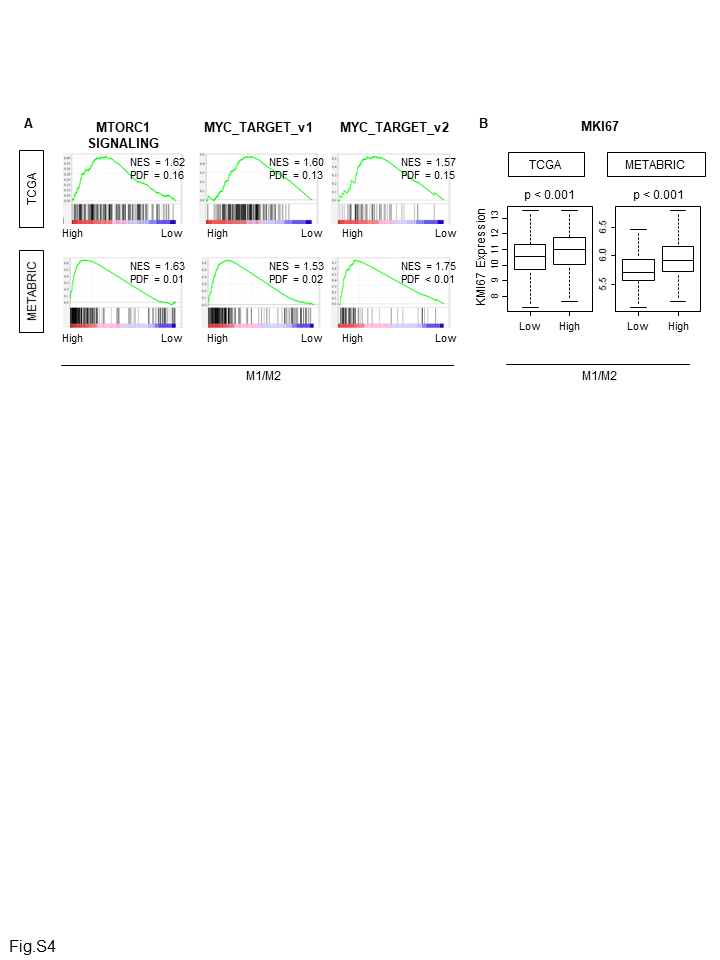


**Supplementary Figure S4**: (A) “M1”/”M2” high tumors were associated with the gene sets related to cell proliferation with only METBRIC cohort. (B) “M1”/”M2” high tumors were associated with Ki-67 transcriptome analysis in both TCGA and METABRIC cohort.


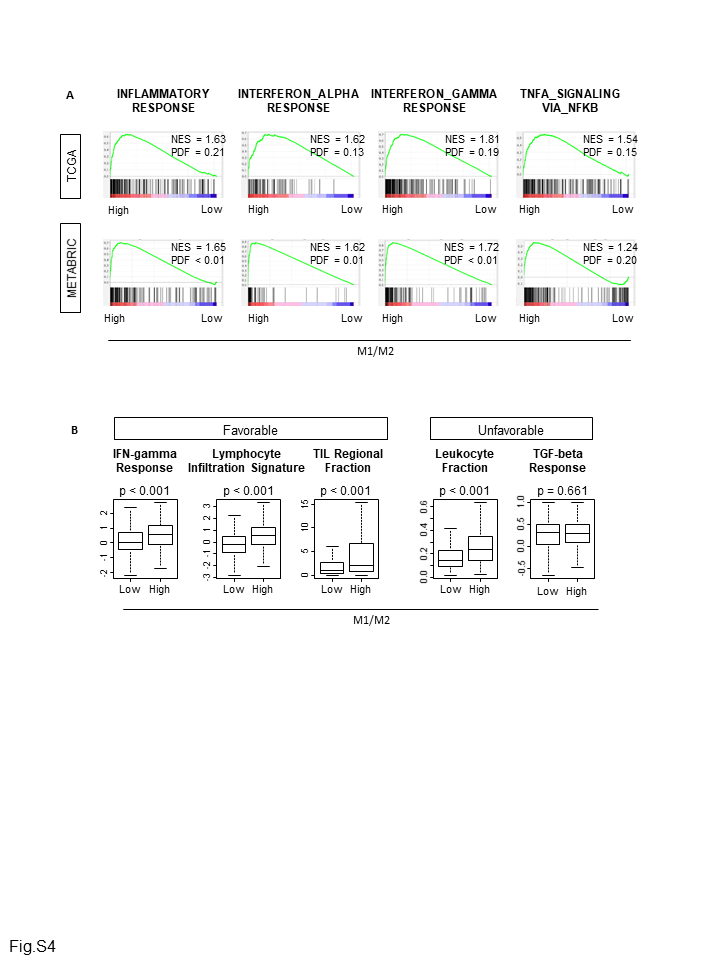


**Supplementary Figure S5**: “M1”/”M2” high tumors enriched the immune associated gene sets in METABRIC only and demonstrated higher immune activity related scores. (A) M1 high tumors enriched the gene sets related immune activity with METABRIC cohort only. (B) High ratio of “M1”/”M2” was associated with higher score of both favorable and unfavorable immune activity related scores with TCGA.


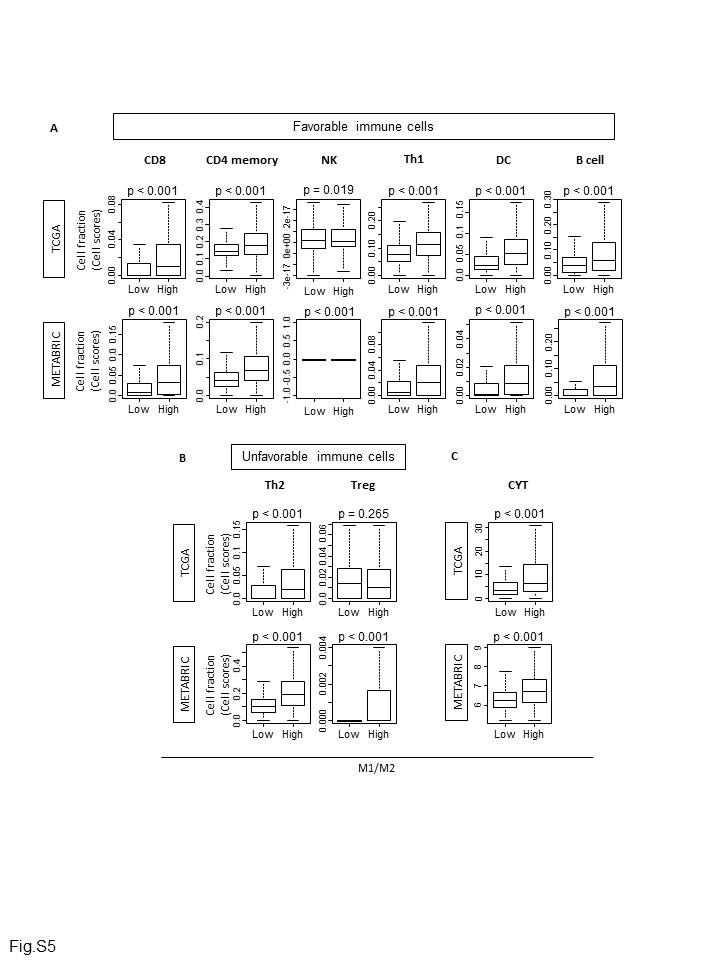


**Supplementary Figure S6**: “M1”/”M2” high tumors demonstrated the infiltration of both favorable and unfavorable immune cells in TCGA and METABRIC cohort.

Th1/2, T-helper 1/2; DC, Dendritic Cell; Treg, regulatory T cell; CYT, cytolytic activity score


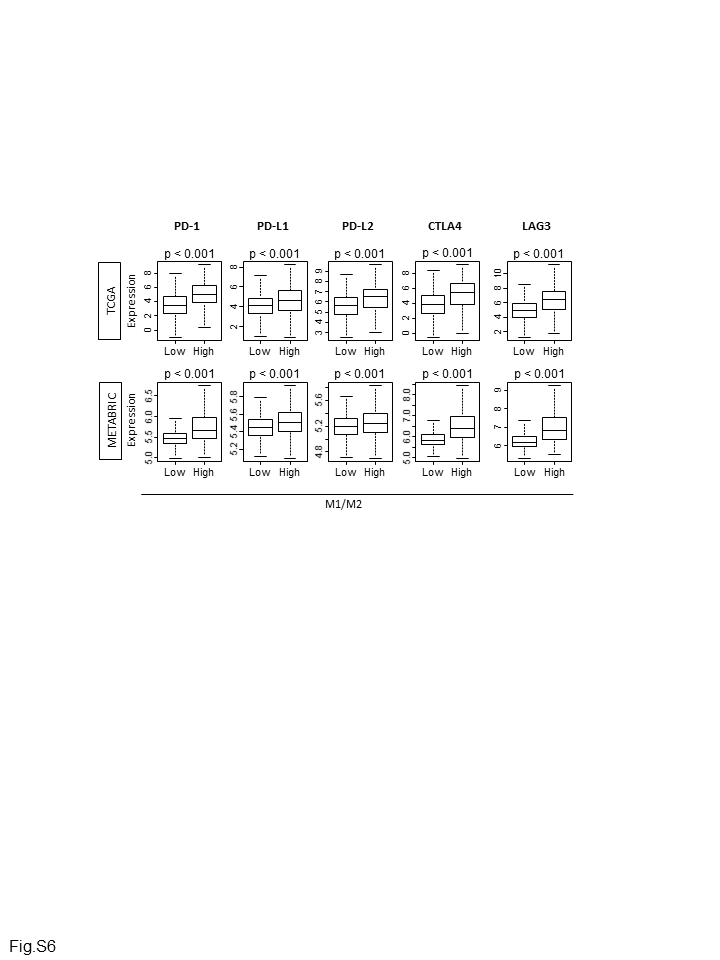


**Supplementary Figure S****7**: “M1”/”M2” high tumors demonstrated the association with T cell exhaustion markers in both TCGA and METABRIC cohort.

PD-1, programmed death-1; PD-L1/2, programmed death ligand 1/2; CTLA4, cytotoxic T-lymphocyte-associated protein 4; LAG3, lymphocyte activation gene 3.

**Supplementary Table S1**: **Gene Name and Title of the M1 macrophage.**

| Gene Name | Gene Title |
| --- | --- |
| ABCD1 | ATP binding cassette subfamily D member 1 |
| ABI1 | abl interactor 1 |
| ABTB2 | ankyrin repeat and BTB domain containing 2 |
| ACP2 | acid phosphatase 2, lysosomal |
| ACTR2 | actin related protein 2 |
| ACTR3 | actin related protein 3 |
| ADAMDEC1 | ADAM like decysin 1 |
| ADCK2 | aarF domain containing kinase 2 |
| ADCY3 | adenylate cyclase 3 |
| ADO | 2-aminoethanethiol dioxygenase |
| ADRA2B | adrenoceptor alpha 2B |
| AFG3L2 | AFG3 like matrix AAA peptidase subunit 2 |
| AGPS | alkylglycerone phosphate synthase |
| ALCAM | activated leukocyte cell adhesion molecule |
| ANXA2 | annexin A2 |
| AP1M2 | adaptor related protein complex 1 subunit mu 2 |
| ARHGEF11 | Rho guanine nucleotide exchange factor 11 |
| ARL8B | ADP ribosylation factor like GTPase 8B |
| ATOX1 | antioxidant 1 copper chaperone |
| ATP6V0C | ATPase H+ transporting V0 subunit c |
| ATP6V1A | ATPase H+ transporting V1 subunit A |
| ATP6V1D | ATPase H+ transporting V1 subunit D |
| ATP6V1E1 | ATPase H+ transporting V1 subunit E1 |
| ATP6V1F | ATPase H+ transporting V1 subunit F |
| ATP6V1H | ATPase H+ transporting V1 subunit H |
| BCAP31 | B cell receptor associated protein 31 |
| BCKDK | branched chain ketoacid dehydrogenase kinase |
| BLVRA | biliverdin reductase A |
| C1QA | complement C1q A chain |
| C1QB | complement C1q B chain |
| C3AR1 | complement C3a receptor 1 |
| CALR | calreticulin |
| CCDC47 | coiled-coil domain containing 47 |
| CCL1 | C-C motif chemokine ligand 1 |
| CCL18 | C-C motif chemokine ligand 18 |
| CCL19 | C-C motif chemokine ligand 19 |
| CCL22 | C-C motif chemokine ligand 22 |
| CCL24 | C-C motif chemokine ligand 24 |
| CCL7 | C-C motif chemokine ligand 7 |
| CCL8 | C-C motif chemokine ligand 8 |
| CCR1 | C-C motif chemokine receptor 1 |
| CD163 | CD163 molecule |
| CD300C | CD300c molecule |
| CD48 | CD48 molecule |
| CD63 | CD63 molecule |
| CD80 | CD80 molecule |
| CD84 | CD84 molecule |
| CECR5 | cat eye syndrome chromosome region, candidate 5 |
| CHIT1 | chitinase 1 |
| CIAO1 | cytosolic iron-sulfur assembly component 1 |
| CLCN7 | chloride voltage-gated channel 7 |
| CLEC4E | C-type lectin domain family 4 member E |
| CLPB | ClpB homolog, mitochondrial AAA ATPase chaperonin |
| CLTC | clathrin heavy chain |
| CMKLR1 | chemerin chemokine-like receptor 1 |
| COQ2 | coenzyme Q2, polyprenyltransferase |
| CORO7 | coronin 7 |
| COX5B | cytochrome c oxidase subunit 5B |
| CSF1 | colony stimulating factor 1 |
| CSF1R | colony stimulating factor 1 receptor |
| CXCL9 | C-X-C motif chemokine ligand 9 |
| CYBB | cytochrome b-245 beta chain |
| CYC1 | cytochrome c1 |
| CYFIP1 | cytoplasmic FMR1 interacting protein 1 |
| CYP19A1 | cytochrome P450 family 19 subfamily A member 1 |
| DAGLA | diacylglycerol lipase alpha |
| DLAT | dihydrolipoamide S-acetyltransferase |
| DNAJC13 | DnaJ heat shock protein family (Hsp40) member C13 |
| DNASE2B | deoxyribonuclease 2 beta |
| DOT1L | DOT1 like histone lysine methyltransferase |
| EMILIN1 | elastin microfibril interfacer 1 |
| EXOC5 | exocyst complex component 5 |
| FAM32A | family with sequence similarity 32 member A |
| FANCE | FA complementation group E |
| FCER1G | Fc fragment of IgE receptor Ig |
| FDX1 | ferredoxin 1 |
| FKBP15 | FKBP prolyl isomerase 15 |
| FOLR2 | folate receptor beta |
| FPR2 | formyl peptide receptor 2 |
| FPR3 | formyl peptide receptor 3 |
| FTL | ferritin light chain |
| GLRX2 | glutaredoxin 2 |
| GP1BA | glycoprotein Ib platelet subunit alpha |
| GPD1 | glycerol-3-phosphate dehydrogenase 1 |
| HAMP | hepcidin antimicrobial peptide |
| HAUS2 | HAUS augmin like complex subunit 2 |
| HEXB | hexosaminidase subunit beta |
| HK3 | hexokinase 3 |
| HSPB7 | heat shock protein family B (small) member 7 |
| HYAL2 | hyaluronidase 2 |
| IFNAR1 | interferon alpha and beta receptor subunit 1 |
| IGSF6 | immunoglobulin superfamily member 6 |
| IL10 | interleukin 10 |
| IL12B | interleukin 12B |
| IL17RA | interleukin 17 receptor A |
| ITGAE | integrin subunit alpha E |
| ITGB1BP1 | integrin subunit beta 1 binding protein 1 |
| KCNJ1 | potassium voltage-gated channel subfamily J member 1 |
| KCNJ5 | potassium voltage-gated channel subfamily J member 5 |
| KCNK13 | potassium two pore domain channel subfamily K member 13 |
| KIFC3 | kinesin family member C3 |
| LAIR1 | leukocyte associated immunoglobulin like receptor 1 |
| LAMP1 | lysosomal associated membrane protein 1 |
| LILRB1 | Leukocyte Immunoglobulin-Like Receptor, Subfamily B, member 1 |
| LILRB4 | Leukocyte Immunoglobulin-Like Receptor, Subfamily B, member 4 |
| LIMD2 | LIM domain containing 2 |
| LONP1 | lon peptidase 1, mitochondrial |
| LONRF3 | LON peptidase N-terminal domain and ring finger 3 |
| MAPK13 | mitogen-activated protein kinase 13 |
| MARCO | macrophage receptor with collagenous structure |
| MFSD7 | major facilitator superfamily domain containing 7 |
| MMP19 | matrix metallopeptidase 19 |
| MRPL12 | mitochondrial ribosomal protein L12 |
| MRPL40 | mitochondrial ribosomal protein L40 |
| MRS2 | magnesium transporter MRS2 |
| MS4A4A | membrane spanning 4-domains A4A |
| MSR1 | macrophage scavenger receptor 1 |
| MT2A | metallothionein 2A |
| MYBPH | myosin binding protein H |
| MYH11 | myosin heavy chain 11 |
| MYO7A | myosin VIIA |
| MYOF | myoferlin |
| MYOZ1 | myozenin 1 |
| NARS | asparaginyl-tRNA synthetase |
| NCAPH | non-SMC condensin I complex subunit H |
| NDUFAF1 | NADH:ubiquinone oxidoreductase complex assembly factor 1 |
| NDUFS2 | NADH:ubiquinone oxidoreductase core subunit S2 |
| NECAP2 | NECAP endocytosis associated 2 |
| NRBP1 | nuclear receptor binding protein 1 |
| OGFR | opioid growth factor receptor |
| OTUD4 | OTU deubiquitinase 4 |
| P2RX7 | purinergic receptor P2X 7 |
| PDCL | phosducin like |
| PHLDB1 | pleckstrin homology like domain family B member 1 |
| PKD2L1 | polycystin 2 like 1, transient receptor potential cation channel |
| PLEKHB2 | pleckstrin homology domain containing B2 |
| PQLC2 | PQ loop repeat containing 2 |
| PRDX1 | peroxiredoxin 1 |
| PTGIR | prostaglandin I2 receptor |
| PTPRA | protein tyrosine phosphatase receptor type A |
| RAB3IL1 | RAB3A interacting protein like 1 |
| RELA | RELA proto-oncogene, NF-kB subunit |
| RNH1 | ribonuclease/angiogenin inhibitor 1 |
| RRP1 | ribosomal RNA processing 1 |
| S100A11 | S100 calcium binding protein A11 |
| S1PR2 | sphingosine-1-phosphate receptor 2 |
| SCAMP2 | secretory carrier membrane protein 2 |
| SDS | serine dehydratase |
| SIGLEC1 | sialic acid binding Ig like lectin 1 |
| SIGLEC7 | sialic acid binding Ig like lectin 7 |
| SIGLEC9 | sialic acid binding Ig like lectin 9 |
| SLAMF8 | SLAM family member 8 |
| SLC11A1 | solute carrier family 11 member 1 |
| SLC1A2 | solute carrier family 1 member 2 |
| SLC25A24 | solute carrier family 25 member 24 |
| SLC31A1 | solute carrier family 31 member 1 |
| SLC6A12 | solute carrier family 6 member 12 |
| SNX3 | sorting nexin 3 |
| SPG21 | SPG21 abhydrolase domain containing, maspardin |
| SPR | sepiapterin reductase |
| SRC | SRC proto-oncogene, non-receptor tyrosine kinase |
| STIP1 | stress induced phosphoprotein 1 |
| STX12 | syntaxin 12 |
| STX4 | syntaxin 4 |
| TBC1D16 | TBC1 domain family member 16 |
| TCEB1 | transcription elongation factor B, polypeptide 1 |
| TDRD7 | tudor domain containing 7 |
| TFEC | transcription factor EC |
| TFRC | transferrin receptor |
| TIE1 | tyrosine kinase with immunoglobulin like and EGF like domains 1 |
| TMEM33 | transmembrane protein 33 |
| TMEM70 | transmembrane protein 70 |
| TMX1 | thioredoxin related transmembrane protein 1 |
| TPP1 | tripeptidyl peptidase 1 |
| TREM2 | triggering receptor expressed on myeloid cells 2 |
| TRIP4 | thyroid hormone receptor interactor 4 |
| TSPO | translocator protein |
| UQCR11 | ubiquinol-cytochrome c reductase, complex III subunit XI |
| USP14 | ubiquitin specific peptidase 14 |
| UTP3 | UTP3 small subunit processome component |
| VIM | vimentin |
| VPS33A | VPS33A core subunit of CORVET and HOPS complexes |
| VSIG4 | V-set and immunoglobulin domain containing 4 |
| WDR11 | WD repeat domain 11 |
| WSB2 | WD repeat and SOCS box containing 2 |
| WTAP | WT1 associated protein |
| ZC3H15 | zinc finger CCCH-type containing 15 |
| ZMPSTE24 | zinc metallopeptidase STE24 |

**Supplementary Table S2: Gene Name and Title of the M2 macrophage.**

| Gene Name | Gene Title |
| --- | --- |
| ABCD1 | ATP binding cassette subfamily D member 1 |
| ACP2 | acid phosphatase 2, lysosomal |
| ACSM5 | acyl-CoA synthetase medium chain family member 5 |
| ADAMDEC1 | ADAM like decysin 1 |
| ADCY3 | adenylate cyclase 3 |
| ADRA2B | adrenoceptor alpha 2B |
| AGGF1 | angiogenic factor with G patch and FHA domains 1 |
| AKR7A2 | aldo-keto reductase family 7, member A2 |
| ALDH9A1 | aldehyde dehydrogenase 9 family member A1 |
| ALG9 | ALG9 alpha-1,2-mannosyltransferase |
| ALK | ALK receptor tyrosine kinase |
| ANGPT4 | angiopoietin 4 |
| ANKFY1 | ankyrin repeat and FYVE domain containing 1 |
| ANXA11 | annexin A11 |
| AP1B1 | adaptor related protein complex 1 subunit beta 1 |
| AQP8 | aquaporin 8 |
| ARFGEF2 | ADP ribosylation factor guanine nucleotide exchange factor 2 |
| ARHGEF11 | Rho guanine nucleotide exchange factor 11 |
| ARSB | arylsulfatase B |
| ATP2A2 | ATPase sarcoplasmic/endoplasmic reticulum Ca2+ transporting 2 |
| ATP6V0A1 | ATPase H+ transporting V0 subunit a1 |
| ATP6V0D1 | ATPase H+ transporting V0 subunit d1 |
| ATP6V1C1 | ATPase H+ transporting V1 subunit C1 |
| ATP6V1D | ATPase H+ transporting V1 subunit D |
| BAIAP2 | BAI1 associated protein 2 |
| BCAP31 | B cell receptor associated protein 31 |
| BTBD1 | BTB domain containing 1 |
| C10orf76 | chromosome 10 open reading frame 76 |
| C16orf62 | chromosome 16 open reading frame 62 |
| CAMP | cathelicidin antimicrobial peptide |
| CANX | calnexin |
| CARD14 | caspase recruitment domain family member 14 |
| CCDC85C | coiled-coil domain containing 85C |
| CCDC88A | coiled-coil domain containing 88A |
| CD52 | CD52 molecule |
| CD63 | CD63 molecule |
| CD81 | CD81 molecule |
| CDS2 | CDP-diacylglycerol synthase 2 |
| CEPT1 | choline/ethanolamine phosphotransferase 1 |
| CLCN7 | chloride voltage-gated channel 7 |
| COL4A3BP | collagen type IV alpha 3 binding protein |
| COMMD9 | COMM domain containing 9 |
| CYFIP1 | cytoplasmic FMR1 interacting protein 1 |
| DHX57 | DExH-box helicase 57 |
| DNASE1L3 | deoxyribonuclease 1 like 3 |
| DNASE2B | deoxyribonuclease 2 beta |
| EFR3A | EFR3 homolog A |
| ELK1 | ETS transcription factor ELK1 |
| EXOC1 | exocyst complex component 1 |
| FDX1 | ferredoxin 1 |
| FGR | FGR proto-oncogene, Src family tyrosine kinase |
| FH | fumarate hydratase |
| FKBP15 | FKBP prolyl isomerase 15 |
| FLT1 | fms related tyrosine kinase 1 |
| FTL | ferritin light chain |
| GABARAP | GABA type A receptor-associated protein |
| GGA1 | golgi associated, gamma adaptin ear containing, ARF binding protein 1 |
| GLB1 | galactosidase beta 1 |
| GORASP1 | golgi reassembly stacking protein 1 |
| GPD1 | glycerol-3-phosphate dehydrogenase 1 |
| GSTO1 | glutathione S-transferase omega 1 |
| GUCA1A | guanylate cyclase activator 1A |
| HADHB | hydroxyacyl-CoA dehydrogenase trifunctional multienzyme complex subunit beta |
| HAMP | hepcidin antimicrobial peptide |
| HEXA | hexosaminidase subunit alpha |
| HEXB | hexosaminidase subunit beta |
| HPS1 | HPS1 biogenesis of lysosomal organelles complex 3 subunit 1 |
| HS3ST2 | heparan sulfate-glucosamine 3-sulfotransferase 2 |
| HSPH1 | heat shock protein family H (Hsp110) member 1 |
| IARS2 | isoleucyl-tRNA synthetase 2, mitochondrial |
| IFNAR1 | interferon alpha and beta receptor subunit 1 |
| IPPK | inositol-pentakisphosphate 2-kinase |
| ITGAX | integrin subunit alpha X |
| KCNJ1 | potassium voltage-gated channel subfamily J member 1 |
| KCNJ5 | potassium voltage-gated channel subfamily J member 5 |
| KCNK13 | potassium two pore domain channel subfamily K member 13 |
| KCTD5 | potassium channel tetramerization domain containing 5 |
| KIAA0196 | KIAA0196 |
| LAIR1 | leukocyte associated immunoglobulin like receptor 1 |
| LAMP1 | lysosomal associated membrane protein 1 |
| LILRA2 | leukocyte immunoglobulin-like receptor, subfamily A (with TM domain), member 2 |
| LILRB4 | leukocyte immunoglobulin-like receptor, subfamily B (with TM and ITIM domains), member 4 |
| LONRF3 | LON peptidase N-terminal domain and ring finger 3 |
| MARCO | macrophage receptor with collagenous structure |
| MFN1 | mitofusin 1 |
| MMP19 | matrix metallopeptidase 19 |
| MRM1 | mitochondrial rRNA methyltransferase 1 |
| MS4A4A | membrane spanning 4-domains A4A |
| MSR1 | macrophage scavenger receptor 1 |
| MTMR14 | myotubularin related protein 14 |
| MYO15A | myosin XVA |
| MYO9B | myosin IXB |
| MYOZ1 | myozenin 1 |
| NAGPA | N-acetylglucosamine-1-phosphodiester alpha-N-acetylglucosaminidase |
| NCAPH | non-SMC condensin I complex subunit H |
| NCKAP1L | NCK associated protein 1 like |
| NDUFB1 | NADH:ubiquinone oxidoreductase subunit B10 |
| NFS1 | NFS1 cysteine desulfurase |
| NOP10 | NOP10 ribonucleoprotein |
| NPR1 | natriuretic peptide receptor 1 |
| OS9 | OS9 endoplasmic reticulum lectin |
| OSBPL11 | oxysterol binding protein like 11 |
| P2RX7 | purinergic receptor P2X 7 |
| PABPC4 | poly(A) binding protein cytoplasmic 4 |
| PDCD6IP | programmed cell death 6 interacting protein |
| PDE1B | phosphodiesterase 1B |
| PEX19 | peroxisomal biogenesis factor 19 |
| PICK1 | protein interacting with PRKCA 1 |
| PLEKHM2 | pleckstrin homology and RUN domain containing M2 |
| POGK | pogo transposable element derived with KRAB domain |
| PQLC2 | PQ loop repeat containing 2 |
| RIN2 | Ras and Rab interactor 2 |
| S100A6 | S100 calcium binding protein A6 |
| SCAMP2 | secretory carrier membrane protein 2 |
| SDCBP | syndecan binding protein |
| SDS | serine dehydratase |
| SLAMF8 | SLAM family member 8 |
| SLC25A24 | solute carrier family 25 member 24 |
| SLC25A46 | solute carrier family 25 member 46 |
| SLC31A1 | solute carrier family 31 member 1 |
| SLC38A7 | solute carrier family 38 member 7 |
| SLC39A1 | solute carrier family 39 member 1 |
| SLC6A12 | solute carrier family 6 member 12 |
| SLC6A7 | solute carrier family 6 member 7 |
| SLC9A6 | solute carrier family 9 member A6 |
| SMG5 | SMG5 nonsense mediated mRNA decay factor |
| SNAPC2 | small nuclear RNA activating complex polypeptide 2 |
| SNX1 | sorting nexin 1 |
| SNX2 | sorting nexin 2 |
| SNX3 | sorting nexin 3 |
| SNX5 | sorting nexin 5 |
| SPG21 | SPG21 abhydrolase domain containing, maspardin |
| STX18 | syntaxin 18 |
| STX4 | syntaxin 4 |
| TAF10 | TATA-box binding protein associated factor 10 |
| TBC1D9B | TBC1 domain family member 9B |
| TFEC | transcription factor EC |
| TMED5 | transmembrane p24 trafficking protein 5 |
| TMEM184C | transmembrane protein 184C |
| TMEM70 | transmembrane protein 70 |
| TMEM9B | TMEM9 domain family member B |
| TNFSF14 | TNF superfamily member 14 |
| TPP1 | tripeptidyl peptidase 1 |
| TREM2 | triggering receptor expressed on myeloid cells 2 |
| TSPO | translocator protein |
| UBXN6 | UBX domain protein 6 |
| UCP3 | uncoupling protein 3 |
| UGP2 | UDP-glucose pyrophosphorylase 2 |
| UNC50 | unc-50 inner nuclear membrane RNA binding protein |
| USF2 | upstream transcription factor 2, c-fos interacting |
| VPS35 | VPS35 retromer complex component |
| VPS53 | VPS53 subunit of GARP complex |
| VSIG4 | V-set and immunoglobulin domain containing 4 |
| VTI1B | vesicle transport through interaction with t-SNAREs 1B |
| WDFY3 | WD repeat and FYVE domain containing 3 |
| XPNPEP2 | X-prolyl aminopeptidase 2 |
| ZC3H3 | zinc finger CCCH-type containing 3 |
| ZCCHC4 | zinc finger CCHC-type containing 4 |
| ZNF219 | zinc finger protein 219 |
